# Use of Different Inclusion Criteria to Compare COVID-19 Hospital Admission Rates by Race and Ethnicity: A Cohort Study

**DOI:** 10.3390/healthcare13040381

**Published:** 2025-02-11

**Authors:** Joshua Longcoy, Zeynep Isgor, Sumihiro Suzuki, Elizabeth Lynch, Heng Wang, David Ansell, Tricia J. Johnson

**Affiliations:** 1 RUSH BMO Institute for Health Equity, Rush University Medical Center, 1700 W. Van Buren St. Suite 126B, Chicago, IL 60612, USA; elizabeth_lynch@rush.edu (E.L.); tricia_j_johnson@rush.edu (T.J.J.); 2 Department of Health Systems Management, Rush University Medical Center, 1700 W. Van Buren St. Suite 126B, Chicago, IL 60612, USA; zeynep_isgor@rush.edu; 3 Department of Family and Preventive Medicine, Rush University Medical Center, 1700 W. Van Buren St. Suite 470, Chicago, IL 60612, USA; sumihiro_suzuki@rush.edu (S.S.); hengwang@uic.edu (H.W.); david_ansell@rush.edu (D.A.); 4 Center for Community Health Equity, Rush University Medical Center, 1700 W. Van Buren St. Suite 470, Chicago, IL 60612, USA; 5 Department of Preventive Medicine, Rush Medical College, 1700 W. Van Buren St. Suite 470, Chicago, IL 60612, USA

**Keywords:** COVID-19, hospitalization, health disparities, racial/ethnic health disparities, inclusion criteria, denominators for prevalence and incidence

## Abstract

**Background/Objectives**: Reports documenting racial disparities in COVID-19 hospitalization rates from electronic medical record data have **used** different sample selection methods. Studies including all individuals with a positive COVID-19 test may be vulnerable to misclassification bias if hospitalization status is not captured for all individuals (i.e., if they went to a non-study hospital). A few studies have used only patients who tested positive in the ED and have found different results. In this study, we explore the implications of using different sets of inclusion criteria for analyses that compare COVID-19 hospital admissions by race and ethnicity. **Methods**: Two separate data sets were created by applying the two different COVID-19 testing inclusion criteria to medical records data from a single academic health system. We used logistic regression to compare the odds of COVID-19 hospitalization across race and ethnicity for each data set and compared our results with previous studies. **Results**: We found that using all positive COVID-19 tests as the study sample resulted in higher odds of hospitalization for Black and Hispanic patients relative to White patients. In contrast, using only patients who tested positive in the ED resulted in higher odds of hospitalization for White patients. These findings matched the findings of other studies. **Conclusions**: Patient inclusion criteria should be considered carefully when comparing results from studies of COVID-19 hospitalization.

## 1. Introduction

Hospitalization is an important indicator of severe COVID-19 [1], and several systematic reviews have reported that Black and Hispanic patients were more likely to be hospitalized for COVID-19 than White patients, possibly suggesting disparities in COVID-19 severity [2,3,4]. However, variations in study inclusion criteria—specifically, variations in the study population used for calculations of prevalence and incidence—complicate the interpretation of findings in these reviews. In the individual studies themselves, some studies included as the study population the number of patients who tested positive for COVID-19 at any testing site, whether symptomatic or asymptomatic, while other studies included only patients who presented to the emergency department (ED) with COVID-19 symptoms. Studies including all individuals with a positive COVID-19 test may be vulnerable to misclassification bias if hospitalization status is not captured for all individuals (i.e., including individuals who were admitted to a non-study hospital). Including only patients testing positive for COVID-19 in the ED of a study hospital may decrease the potential for misclassification bias as those patients would be more likely to be hospitalized at the study hospital.

Such differences in inclusion criteria are not uncommon in research and pose a limited threat to validity when results are consistent across studies. However, when results are different across studies, methodological differences may account for the inconsistency. Our previously published study of patients who sought care for COVID-19 symptoms in the ED showed results inconsistent with the vast majority of the existing literature. While the majority of studies examining patients with a positive COVID-19 test have indicated higher hospitalization rates for Black and Hispanic individuals, we found in a previous study that White patients were more likely to be admitted to the hospital than Black patients [5]. We hypothesized that differences in sample inclusion criteria would explain this conflicting finding. Thus, the aim of this study was to compare racial and ethnic differences in COVID-19 hospitalization at a single urban academic health system with a diverse patient population using two sets of patient inclusion criteria: all patients with a positive COVID-19 test (from any testing site within the health system) versus patients who tested positive for COVID-19 in the emergency department.

## 2. Methods

### 2.1. Sample

We obtained data from the EMR of an academic health system in the Chicago, Illinois, area for all patients aged 18 years or older with a positive test for COVID-19 between March 2020 and April 2021. Patients who were hospitalized in inpatient rehabilitation, inpatient psychiatry, hospice, or labor and delivery, after testing positive for COVID-19 were excluded from the analysis to eliminate patients who sought hospital care for symptoms or diagnoses other than COVID-19 since they were likely to have been admitted to the hospital independent of a positive COVID-19 test.

Since all the data came from the same health system, the word “location” in this study refers to testing location/site within the same health system. We defined three patient cohorts for analysis. Cohort 1 included all patients in the system with a positive COVID-19 test (referred to as Cohort 1 or “All positive tests”). Patients in Cohort 1 were then classified based on the location of their first positive COVID-19 test: individuals who first tested positive for COVID-19 in the hospital’s ED (Cohort 2 or “ED positive tests”) and individuals who first tested positive for COVID-19 in any location outside the ED (Cohort 3 or “outside the ED positive tests”). An individual who tested positive at an outpatient location within the health system other than the ED and was tested again in the hospital’s ED was placed in Cohort 3, provided they sought care in the ED within 7 days of the first positive test. For example, if a patient tested positive in a PCP clinic and then sought care in the ED, where the patient was tested again, then this patient would be in Cohort 3 since the test in the ED was a duplicate. Table 1 summarizes the cohorts.

Cohort 1 is comparable to other studies that included all positive tests regardless of location. Cohort 2 and Cohort 3 were two mutually exclusive cohorts representing patients with a positive test in the ED and outside the ED, respectively. Although the main comparison was between Cohort 1 and Cohort 2, Cohort 3 was created specifically to compare the characteristics of individuals who first tested positive in the ED with those who first tested positive elsewhere. The academic health system’s institutional review board approved this study.

### 2.2. Variables

Using the EMR, we followed each patient who tested positive for 2 weeks after the testing date to determine if they were hospitalized. The primary outcome was hospitalization within 7 days after the first positive COVID-19 test within the academic health system. Patients who were transferred from the ED to an observation unit and subsequently admitted to an inpatient hospital unit were classified as admitted.

Patient race and ethnicity were classified based on a self-identified field in the EMR. We limited our analysis to patients who identified as non-Hispanic Black (Black), Hispanic, or non-Hispanic White (White) to compare our results with other published studies that primarily limited their analyses to these three groups. Therefore, we did not analyze data from patients in other racial and ethnic groups (n = 1850) or with missing race or ethnicity information (n = 1372).

The following variables were compared between Cohorts 2 and 3 and/or included as covariates in our analysis: patient age, sex (male or female), insurance (commercial, Medicaid, Medicare, or uninsured), history of care (i.e., whether the patient had an encounter with a primary care provider [PCP] within the hospital system in the 24 months before testing positive, had any ED encounter or hospitalization within the hospital system within the 12 months before testing positive, or had any PCP in the hospital system or elsewhere on file), the month and year of the positive COVID-19 test, and COVID-19 test location. The test location of the patient’s first positive COVID-19 test was classified as drive-through, ED, employee testing, primary care physician/specialist clinic (e.g., obstetrics/gynecology, cancer), urgent/convenient care, and other outpatient settings (including ambulatory surgery and homeless shelters).

### 2.3. Statistical Analyses

We calculated frequencies (percentages) for each variable within each cohort: patients tested outside the ED (Cohort 3), patients who sought tests in the ED (Cohort 2), and all patients with a positive test (Cohort 1). We used chi-squared tests to compare patient characteristics between Cohort 2 and Cohort 3 and to compare patient characteristics by race and ethnicity within each of the cohorts.

Three logistic regression models were then constructed to examine the relationship between race and ethnicity and COVID-19 hospitalization for each cohort. For each cohort, we constructed a model that included race and ethnicity as a single independent variable and a model adjusted for race and ethnicity, sex, insurance, and age. We included only these covariates to ensure comparability with other published studies. All models were adjusted for the month and year the patient tested positive to control for temporal patterns related to the progression of the pandemic. The predictive accuracy of each model was assessed using the area under the receiver operating characteristic curve, and goodness-of-fit was evaluated with the Hosmer–Lemeshow test. All statistical analyses were performed using SAS version 9.4 (SAS Institute, Cary, NC, USA).

## 3. Results

### Sample Characteristics

In total, 19,421 unique patients tested positive for COVID-19 during the study period. Of these patients, 7806 (40%) tested positive in the medical center’s ED (Table 2). About 18% of the patients in Cohort 1 were admitted. While 43% of the patients tested in the ED (Cohort 2) were admitted, less than 2% of the patients tested outside the ED (Cohort 3) were admitted. About half of the patients who tested positive anywhere in the system had commercial insurance and were younger. Patients who tested positive in the ED were older and more likely to have Medicare or Medicaid insurance compared to patients who tested positive outside the ED. Additionally, fewer patients who tested positive in the ED had a PCP on file (80% vs. 87%) or had a PCP visit in the prior 24 months (28% vs. 38%). Patients who tested positive in the ED were more likely to have had any encounter in the health system (i.e., ED, PCP, outpatient, or inpatient encounter) (67% vs. 42%).

Of all patients who tested positive, 32% were Black, 44% were Hispanic, and 24% were White. Black patients were more likely to be tested in the ED (54%) than Hispanic patients (38%) or White patients (26%).

Of patients who first tested positive in the ED, Hispanic patients were least likely to be admitted (39%), while Black and White patients were equally likely to be admitted (60%). In this cohort (Cohort 2), White patients were older than Black and Hispanic patients (Table 3). About 10% of Black patients, 27% of Hispanic patients, and 6% of White patients in Cohort 2 were uninsured. Of patients who tested positive outside of the ED, 3% of Black patients, 2% of Hispanic patients, and 2% of White patients subsequently sought care within the ED. Black patients who sought care in the ED after testing positive elsewhere were less likely to be admitted than Hispanic or White patients (Black: 37%, Hispanic: 78%, White: 83%) (see Supplemental Figure S1).

In patients who tested positive at any location (Cohort 1), after adjusting for sex, age, and insurance, Black patients had 1.88 times higher odds of hospitalization than White patients (95% CI: 1.67, 2.13; Table 4). In contrast, among patients who tested positive in the ED (Cohort 2), Black patients were significantly less likely to be admitted to the hospital than White patients (OR = 0.69; 95% CI: 0.58, 0.81). Similarly, among Cohort 1 patients, Hispanic patients were 1.52 times more likely to be hospitalized than White patients (95% CI: 1.35, 1.72), yet Hispanic patients who tested positive in the ED (Cohort 2) were significantly less likely to be hospitalized than White patients (OR = 0.80, 95% CI: 0.68, 0.94). Among those who tested positive outside the ED (Cohort 3), race and ethnicity were not associated with the odds of hospitalization.

## 4. Discussion

This study compared the odds of COVID-19 hospitalization by race and ethnicity for a single academic health system using two sets of inclusion criteria: (1) all positive tests recorded in the system (Cohort 1) and (2) only patients who tested positive in the ED (Cohort 2). Our findings revealed that Black and Hispanic patients were more likely than White patients to be hospitalized in the cohort of all positive tests (Cohort 1), while White patients were more likely to be hospitalized than Black or Hispanic patients in the cohort of patients with positive tests in the ED (Cohort 2). This divergence suggests potential disparities in healthcare access and utilization that warrant future exploration. For example, in the current study, Black and Hispanic patients who were tested in the ED may have been less likely than White patients to be hospitalized because they had more limited access to alternative testing sites and, therefore, were more likely than Whites to go to the ED with less severe symptoms that did not require hospitalization. Prior studies have found that Black and Hispanic individuals are more likely than White individuals to use the ED for non-emergent healthcare needs [6,7,8].

### 4.1. Comparison with Findings from Published Literature

In our review of studies examining COVID-19 hospitalizations that used EMR data (see Supplemental Table S1 for search criteria), we identified 15 studies that reported odds of hospitalization by race and ethnicity. Among these studies, 13 included patients with a positive test from any test location, like our Cohort 1 (13 comparing Black and White patients; 9 comparing Hispanic and White patients), and 2 studies included only patients who tested positive in the ED like our Cohort 2 (1 study comparing Black and White patients; both comparing Hispanic and White patients). In the studies that included all positive tests, seven found significantly higher odds of hospitalization for Black patients compared to White patients [9,10,11,12,13,14,15], while six studies found no difference (Supplemental Table S2) [16,17,18,19,20,21]. The single ED-specific study comparing Black and White patients found no difference in the odds of hospitalization [22].

Among the nine studies comparing Hispanic and White patients with positive tests from any location, six studies found higher odds of admission for Hispanic patients [9,10,12,14,18,20], one found no difference [19], and two found lower odds of hospitalization for Hispanic patients [16,21]. For ED-specific studies, of the two comparing Hispanic vs. White patients, one study found no difference in the odds of hospitalization [22], while the other study found lower odds of hospitalization for Hispanic patients [23].

### 4.2. EMR Data and Misclassification Bias

During the height of the COVID-19 pandemic, academic health systems established test locations throughout their communities, many collaborating extensively with public health authorities to ensure testing accessibility regardless of hospital geographic catchment area [24,25]. These community-based test locations often collected minimal patient information and served individuals regardless of whether they had established care within the system. Simultaneously, long ED wait times may have led individuals to seek care at any available health system. Because EMR data are limited to within-system utilization, studies of COVID-19 hospitalization rates among all positive cases (including community-based testing locations) may underestimate the actual hospitalization rates by missing admissions to non-study hospitals. That is, these analyses are vulnerable to misclassification bias because only hospitalizations at the study hospital(s) are known, and hospitalizations at other hospitals would not have been represented in the data set. Therefore, studies using samples including all positive COVID-19 tests may underestimate hospitalization rates. This may be less likely for studies that include only positive tests from the health system ED because individuals were likely to have been directly admitted to the hospital rather than to a non-study hospital. In the data used for this study, we saw that patients tested outside the ED were less likely to be admitted than patients tested in the ED (Supplemental Table S3). This is important to note since data from outpatient settings that are within the same healthcare system can still be downloaded for researchers to analyze. However, it might not always be advantageous to download everything (i.e., all patients testing positive for COVID-19) in the system [26]. Caution should be used when selecting the inclusion and exclusion criteria in studies using EMR data.

### 4.3. Variations in Healthcare Ecosystems Across Studies

Variations in healthcare ecosystems across studies may also explain variations in the magnitude and direction of findings regarding disparities in COVID-19 hospitalizations. Differences in the number and geographic locations of community-based testing sites and outpatient clinics offering COVID-19 testing are one of several factors that may have led to variations in the characteristics of patients who sought care for COVID-19 in the ED versus other testing sites. Another factor is the socioeconomic composition of the health system’s geographic catchment area. For example, hospital systems serving larger uninsured populations or with limited primary care access likely experienced higher non-emergent ED utilization for COVID-19 [27]. Conversely, systems serving predominantly insured populations and/or with extensive primary care networks may have had fewer low-severity patients seeking COVID-19-related care in the ED. For example, we found significant differences in the characteristics of patients who tested positive in the ED (Cohort 2) versus outside of the ED (Cohort 3), even after accounting for race and ethnicity. Patients who tested positive outside the hospital were more likely to have commercial insurance, were younger on average, and were more likely to have seen a PCP in the prior 24 months compared to patients who sought care in the ED (Cohort 2; see Table 3). These differences may have fundamentally affected the extent to which less severely ill patients accessed COVID-19 testing in the ED. Thus, not accounting for these underlying population characteristics and disparities in healthcare access may yield non-comparable results. In future studies, it is important that authors explain the healthcare ecosystem, including the socioeconomic characteristics of the catchment area and the number of other hospital systems with overlapping catchment areas.

### 4.4. Limitations

Though this study sheds new light on how differences in inclusion criteria may explain conflicting findings in studies of COVID-19 hospitalization, there are several limitations. First, we did not conduct a full systematic review because three systematic reviews had already reported differences in hospital admission rates between racial and ethnic groups [2,3,4]. Second, our review of inclusion criteria in the previously published studies was limited to the information reported, and information about hospital catchment areas and community demographic characteristics was often limited. Thus, we may have omitted important information relevant to comparisons across studies. However, by using data from one hospital system for our analysis, we could compare results across different inclusion criteria while holding catchment area and other factors constant, providing a possible explanation for differences by race and ethnicity among published studies. Third, race or ethnicity data were missing for 11% of the individuals who tested outside the ED in our sample. Collecting complete data on race and ethnicity is an area for improvement and was heavily promoted in the latter part of 2020 [28]. Lastly, since this study was conducted within a single health system, the results may not be generalizable. However, the majority (9) of the 15 studies we found comparing COVID-19 hospitalizations for Black, Hispanic, and White patients were from a single health system. Nonetheless, by comparing our results to currently published results, we believe this strengthens the conclusions of our study.

## 5. Conclusions

This study highlights important considerations for using hospital system-level data to study racial and ethnic disparities in COVID-19 hospitalizations and describes how different results may arise for studies of patients who tested positive at any test location versus in the ED. Our findings suggest that test location is important because individuals testing positive for COVID-19 at different physical locations might not have an equal likelihood of hospital admission. The type of test location (e.g., outpatient vs. ED) may also be important to consider. Sensitivity analyses that use different inclusion criteria may help reveal other potential selection biases that may occur.

## Figures and Tables

**Table 1:** Summary of the cohorts. All data came from the same academic health system.

	Name	Description
Cohort1	All positive tests	All positive COVID-19 tests in the EMR system regardless of the test location.
Cohort2	ED positive tests	Patients who tested positive for COVID-19 for the first time in the hospital system’s ED; included all encounters with COVID-19 as a diagnosis code.
Cohort3	Outside the ED positive tests	Patients who tested positive for COVID-19 outside of the ED and sought care in the ED within 7 days after the positive test.

**Table 2:** Sample characteristics: patients in a Chicago-area academic health system who tested positive for COVID-19 from March 2020 through April 2021 (N = 19,241).

Variable, n (%)	Cohort 1 (All Positive Tests)	Cohort 2 (ED Positive Tests)	Cohort 3 (Outside the ED Positive Tests)
	N = 19,421	N = 7806	N = 11,615
Age			
18–29	4333 (22.3)	1427 (18.3)	2906 (25.0)
30–39	4078 (21.0)	1297 (16.6)	2781 (23.9)
40–49	3623 (18.7)	1338 (17.1)	2285 (19.7)
50–59	3264 (16.8)	1335 (17.1)	1929 (16.6)
60–69	2205 (11.4)	1091 (14.0)	1114 (9.6)
70–79	1271 (6.5)	813 (10.4)	458 (3.9)
80–89	510 (2.6)	384 (4.9)	126 (1.1)
90+	137 (0.7)	121 (1.6)	16 (0.1)
Male	8364 (43.1)	3614 (46.3)	4750 (40.9)
Race and ethnicity			
Black	6137 (31.6)	3334 (42.7)	2803 (24.1)
Hispanic	8629 (44.4)	3238 (41.5)	5391 (46.4)
White	4655 (24.0)	1234 (15.8)	3421 (29.5)
Insurance			
Commercial	9581 (49.3)	2632 (33.7)	6949 (59.8)
Medicaid	3484 (17.9)	2010 (25.8)	1474 (12.7)
Medicare	2821 (14.5)	1877 (24.1)	944 (8.1)
Uninsured	3534 (18.2)	1287 (16.5)	2247 (19.4)
ED encounter	8019 (41.3)		221 (1.9)
Has PCP	16,098 (83.9)	6186 (80.0)	9912 (86.6)
Had PCP visit in prior 24 months	6588 (33.9)	2172 (27.8)	4416 (38.0)
Had any encounter in prior 12 months	10,106 (52.0)	5233 (67.0)	4873 (42.0)
Test locations			
Drive-through	6311 (32.5)		
ED	7806 (40.2)		
Employee testing	237 (1.2)		
PCP/Specialist clinic	245 (1.3)		
Urgent/Convenient care	1425 (7.3)		
Other outpatient	3397 (17.5)		
Admitted	3495 (18.0)	3318 (42.5)	177 (1.5)

Cohort 1 includes all COVID-19 positive tests that occurred within the academic health system, including tests that occurred in and out of the ED. Cohort 2 includes all ED encounters with COVID-19 as a diagnosis code within the health system. Cohort 3 includes all COVID-19 positive tests that occurred within the hospital system but outside the ED, if within 7 days before an ED encounter. Cohort 2 was significantly different (*p*-value < 0.05) from Cohort 3 in every variable. Abbreviations: ED, emergency department; PCP, primary care provider.

**Table 3:** Sample characteristics by race and ethnicity for Cohort 2 (ED positive tests) and Cohort 3 (outside the ED positive tests).

	Cohort 2 (ED Positive Tests)			Cohort 3 (Outside the ED Positive Tests)		
Variable, n (%)	Black n = 3334	Hispanic n = 3238	White n = 1234	Black n = 2803	Hispanic n = 5391	White n = 3421
Age						
18–29	**676 (20.3** **)**	**641 (19.8)**	110 (8.9)	**518 (18.5** **)**	**1579 (29.3)**	809 (23.7)
30–39	**631 (18.9** **)**	**533 (16.5)**	133 (10.8)	**659 (23.5** **)**	**1378 (25.6)**	744 (21.8)
40–49	**561** **(16.8)**	**653 (20.2)**	124 (10.1)	**597 (21.3** **)**	**1110 (20.6)**	578 (16.9)
50–59	**517 (15.5** **)**	**618 (19.1)**	200 (16.2)	**518 (18.5** **)**	**793 (14.7)**	618 (18.1)
60–69	**466 (14.0** **)**	**420 (13.0)**	205 (16.6)	**342 (12.2** **)**	**364 (6.8)**	408 (11.9)
70–79	**303 (** **9.1)**	**246 (7.6)**	264 (21.4)	**130 (** **4.6)**	**125 (2.3)**	203 (5.9)
80–89	**141 (4.2** **)**	**110 (3.4)**	133 (10.8)	**33 (1.2** **)**	**37 (0.7)**	56 (1.6)
90+	**39 (1** **.2)**	**17 (0.5)**	65 (5.3)	**6 (0.2** **)**	**5 (0.1)**	5 (0.2)
Male	**1374 (41.2)**	1630 (50.3)	610 (49.4)	**903 (32.2** **)**	2389 (44.3)	1458 (42.6)
Insurance						
Commercial	**1026 (30.8** **)**	**1142 (35.3)**	464 (37.6)	**1553 (55.4** **)**	**2866 (53.2)**	2530 (74.0)
Medicaid	**1131 (33.9** **)**	**716 (22.1)**	163 (13.2)	**535 (19.1** **)**	**746 (13.8)**	193 (5.6)
Medicare	**837 (25.1** **)**	**508 (15.7)**	532 (43.1)	**325 (11.6** **)**	**254 (4.7)**	365 (10.7)
Uninsured	**340 (10.2** **)**	**872 (26.9)**	75 (6.1)	**390 (13.9** **)**	**1524 (28.3)**	333 (9.7)
ED encounter				70 (2.5)	85 (1.6)	66 (1.9)
Has PCP	**2622 (79.4** **)**	**2511 (78.3** **)**	1053 (86.0)	2404 (87.0)	**4547 (85.6)**	2961 (87.9)
Had PCP visit in prior 24 months	**1009 (30.3** **)**	830 (25.6)	333 (27.0)	1278 (45.6)	**1565 (29.0)**	1573 (46.0)
Had any encounter in prior 12 months	**2304 (69.** **1)**	**2013 (62.2** **)**	916 (74.2)	1442 (51.4)	**1727 (32.0)**	1704 (49.8)
Admitted	**1319 (39.6)**	**1268 (39.2)**	731 (59.2)	53 (1.9)	70 (1.3)	54 (1.6)

**Bold** numbers indicate *p*-value < 0.05 in comparison to White patients in the same cohort. Abbreviations: ED, emergency department; PCP, primary care physician.

**Table 4:** Associations between race and ethnicity and hospital admission by patient cohort.

	Cohort 1 (All Positive Tests) N = 19,421	Cohort 2 (ED Positive Tests) n = 7806	Cohort 3 (Outside the ED Positive Tests) n = 11,615
Model 1			
White	1.0	1.0	1.0
Black	**1.59 (1.43, 1.76)**	**0.38 (0.33, 0.44)**	1.25 (0.85, 1.86)
Hispanic	0.96 (0.87, 1.06)	**0.41 (0.35, 0.47)**	0.88 (0.61, 1.28)
Model 2			
White	1.0	1.0	1.0
Black	**1.88 (1.67, 2.13)**	**0.69 (0.58, 0.81)**	1.28 (0.85, 1.91)
Hispanic	**1.52 (1.35, 1.72)**	**0.80 (0.68, 0.94)**	1.39 (0.94, 2.04)

Model 1: Adjusted only for race and ethnicity and the month/year the patient tested positive. Model 2: Adjusted for race and ethnicity, sex, insurance, age, and the month/year the patient tested positive. **Bold** numbers indicate *p*-value < 0.05 in comparison to White patients within the same cohort. Odds ratios are shown with 95% CI.

## Data Availability

The anonymized data analyzed in this study are available upon reasonable request from the corresponding author.

## Supplementary Materials

The following supporting information can be downloaded at https://www.mdpi.com/article/10.3390/healthcare13040381/s1, Supplemental Table S1: Search terms for the literature review; Supplemental Table S2: Summary of included studies; Supplemental Table S3: Sample characteristics: hospital patients positive for COVID-19 from March 2020 through April 2021 (N = 19,241) who were admitted vs. not admitted; Supplemental Figure S1: Flowchart of the percentage of COVID-19 positive patients admitted by race and ethnicity.
