# Supplementary material for: Use of Different Inclusion Criteria to Compare COVID-19 Hospital Admission Rates by Race and Ethnicity: A Cohort Study
Source: Healthcare (Basel). 2025 Feb 11;13(4):381. doi: 10.3390/healthcare13040381 (PMC11855206; doi:10.3390/healthcare13040381)
Supplement: Supplementary file 1 [file healthcare-13-00381-s001.zip › healthcare-3331441-supplementary.pdf]

Supplemental Table S1. Search Terms for Literature Review

<u>**PubMed**</u><br><br>**("COVID-19" [Mesh] OR "SARS-CoV-2"[Mesh] OR "severe acute respiratory syndrome coronavirus 2" [Supplementary Concept] OR "covid-19"[tiab] OR "covid 19"[tiab] OR covid19[tiab] OR "sars-cov-2"[tiab] OR "sars cov 2"[tiab] OR "2019 novel coronavirus"[tiab] OR "2019-ncov"[tiab] OR "2019 ncov"[tiab]) AND ("Racial Groups"[Mesh] OR "Race Factors"[Mesh] OR "Ethnicity"[Mesh] OR race*[tiab] OR racial[tiab] OR ethnic*[tiab] OR "skin color"[tiab]) AND ("Hospitalization"[Mesh] OR "Intensive Care Units"[Mesh] OR hospital*[tiab] OR admit*[tiab] OR admission*[tiab] OR "intensive care unit"[tiab] OR "intensive care units"[tiab])** = 1,406 results<br><br>**("COVID-19" [Mesh] OR "SARS-CoV-2"[Mesh] OR "severe acute respiratory syndrome coronavirus 2" [Supplementary Concept] OR "covid-19"[tiab] OR "covid 19"[tiab] OR covid19[tiab] OR "sars-cov-2"[tiab] OR "sars cov 2"[tiab] OR "2019 novel coronavirus"[tiab] OR "2019-ncov"[tiab] OR "2019 ncov"[tiab]) AND ("Racial Groups"[Mesh] OR "Race Factors"[Mesh] OR "Ethnicity"[Mesh] OR race*[tiab] OR racial[tiab] OR ethnic*[tiab] OR "skin color"[tiab]) AND ("Hospitalization"[Mesh] OR "Intensive Care Units"[Mesh] OR hospital*[tiab] OR admit*[tiab] OR admission*[tiab] OR "intensive care unit"[tiab] OR "intensive care units"[tiab]) AND ("COVID-19 Testing"[Mesh] OR "Point-of-Care Testing"[Mesh] OR "Direct-To-Consumer Screening and Testing"[Mesh] OR "Diagnosis"[Mesh] OR test*[tiab] OR screen*[tiab] OR diagnos*[tiab])** = 822 results<br><br>**("COVID-19" [Mesh] OR "SARS-CoV-2"[Mesh] OR "severe acute respiratory syndrome coronavirus 2" [Supplementary Concept] OR "covid-19"[tiab] OR "covid 19"[tiab] OR covid19[tiab] OR "sars-cov-2"[tiab] OR "sars cov 2"[tiab] OR "2019 novel coronavirus"[tiab] OR "2019-ncov"[tiab] OR "2019 ncov"[tiab]) AND ("Racial Groups"[Mesh] OR "Race Factors"[Mesh] OR "Ethnicity"[Mesh] OR race*[tiab] OR racial[tiab] OR ethnic*[tiab] OR "skin color"[tiab]) AND ("COVID-19 Testing"[Mesh] OR "Point-of-Care Testing"[Mesh] OR "Direct-To-Consumer Screening and Testing"[Mesh] OR "Diagnosis"[Mesh] OR test*[tiab] OR screen*[tiab] OR diagnos*[tiab])** = 1974 results
<u>**Scopus**</u><br><br>**TITLE-ABS-KEY("covid-19" OR "covid 19" OR covid19 OR "sars-cov-2" OR "sars cov 2" OR "2019 novel coronavirus" OR "2019-ncov" OR "2019 ncov") AND TITLE-ABS-KEY(race* OR racial OR ethnic* OR "skin color") AND TITLE-ABS-KEY(hospital* OR admit* OR admission* OR "intensive care unit" OR "intensive care units")** = 1,710 results
<u>**CINAHL**</u><br><br>**(MH "COVID-19" OR "covid-19" OR "covid 19" OR covid19 OR "sars-cov-2" OR "sars cov 2" OR "2019 novel coronavirus" OR "2019-ncov" OR "2019 ncov") AND (MH "Race Factors" OR MH "Ethnic Groups" OR race* OR racial OR ethnic* OR "skin color") AND (MH "Hospitalization" OR MH "Intensive Care Units" OR hospital* OR admit* OR admission* OR "intensive care unit" OR "intensive care units")** = 526 results

Supplementary Table S2. Summary of Included Studies

Study	Setting	Date range	Study population	Inclusion criteria	Black vs. White results	Hispanic vs. White results	Covariates
Azar, 2021	Northern California, rural and urban; Sutter Health System	Jan 20, 2020, to Aug 31, 2020	Anyone who accessed care during study period	Positive for COVID-19	SIP: 2.08 (1.32, 3.23) Post-SIP: 1.55 (1.23, 1.96)	SIP: 1.77 (1.31, 2.40) Post-SIP: 1.58 (1.37, 1.83)	
Dai, 2021	California, Oregon, Washington; Providence St. Joseph Health System	Mar 2020 to Jan 2021	Patients from California, Oregon, and Washington who were tested for COVID-19	Positive for COVID-19	aOR: 0.98 (0.87, 1.1)	aOR: 0.85 (0.8, 0.9)	Race and ethnicity, age, square of age sex, insurance, neighborhood median income, crowded housing, LEP, minority, CCI, hypertension, obesity
Escobar, 2021	Northern California; Kaiser Permanente Northern California	Feb 2020 to May 2020	Patients who were members during study period		aOR: 1.47 (1.03, 2.09)	aOR: 1.42 (1.11, 1.82)	Age, sex, NDI, comorbidities, race and ethnicity
Gerwen, 2020	New York City; NYC Health System	Mar 1, 2020, to Apr 1, 2020	Anyone testing positive	Positive for COVID-19	aOR: 1.08 (0.86, 1.35)		Age, sex, race, BMI, smoking status, comorbidities
Gu, 2020	Southeast Michigan; University of Michigan Health System	Mar 10, 2020, to Apr 22, 2020	Patients with a COVID-19 test result at Michigan Medical School	Positive for COVID-19	aOR: 1.72 (1.15, 2.58)		Age, sex, race and ethnicity, NDI, comorbidity score
Ingraham, 2021	Minnesota; pooled EHR data across systems including 12 hospitals and 69 clinics	Mar 4, 2020, to Aug 19, 2020	Only patients positive for COVID-19	Positive for COVID-19	aOR: 1.50 (1.15, 1.94)	aOR: 3.80 (2.72, 5.30)	Age, sex, Elixhauser Comorbidity score, relationship status, rurality/urbanity
Jacobson, 2021	Northern California; Contra Costa Regional Medical Center, County Public	Feb 28, 2020, to Mar 4, 2021	Patients impaneled at a northern California regional	Positive for COVID-19	aOR: 1.29 (0.80, 2.08)	aOR: 3.23 (2.06, 5.08)	Race and ethnicity, asthma, cancer, pain, diabetes, COPD,

Study	Setting	Date range	Study population	Inclusion criteria	Black vs. White results	Hispanic vs. White results	Covariates
	Hospital and affiliated health centers		medical center and enrolled in the county Medicaid managed care plan				hypertension, Age, BMI, sex, median income, average household size, share employed, share uninsured, share cash assistance, share social security, share receiving SNAP, share detached single family home
Ogedegbe, 2020	New York City; New York University health system	Mar 1, 2020, to Apr 8, 2020	All patients who were tested in the New York University health system	Positive for COVID-19	aOR: 0.9 (0.7, 1.1)	aOR: 1.1 (0.9, 1.3)	Age, sex, obesity, smoking, diabetes, hypertension, hyperlipidemia, coronary artery disease, chronic kidney disease, heart failure, COPD, asthma, cancer
Petrilli, 2020	New York City; NYU Langone Health	Mar 1, 2020, to Apr 8, 2020	Anyone testing positive	Positive for COVID-19	aOR: 0.81 (0.65, 1.01)	aOR: 1.63 (1.35, 1.97)	Comorbidities, BMI, smoking, sex, age, week number
Poulson, 2021	United States; CDC Surveillance Review and Response Group	Apr 5, 2020, to May 18, 2020	All Black and White patients positive for COVID-19	Positive for COVID-19	aRR: 1.42 (1.40, 1.44)		Race and ethnicity, age, sex, comorbidities
Price-Haywood, 2020	Louisiana; Ochsner Health Population	Mar 1 to Apr 11, 2020	Patients seen at an Ochsner Health facility positive for COVID-19	Positive for COVID-19	aOR: 2.35 (1.97, 2.80)		Age, sex

Study	Setting	Date range	Study population	Inclusion criteria	Black vs. White results	Hispanic vs. White results	Covariates
Qeadan, 2021	United States ; Cerner Real-World Data	Jan 2020 to Jun 2020	Patients with encounters associated with a diagnosis of or a recent positive lab test for COVID-19	Positive for COVID-19	aOR: 1.02 (0.95, 1.08)	aOR: 0.81 (0.77, 0.86)	Age, sex, race and ethnicity, insurance, Elixhauser Comorbidity score
Valenzuela, 2020	60 miles outside of Manhattan, suburban Suffolk county, Long Island, New York	Mar 7, 2020, to May 23, 2020	Only patients who presented at the ED positive for COVID-19	Positive for COVID-19 in the ED		aOR: 0.69 (0.52-0.92)	Sex, age, temp, tachypnea, oxygen, any exposure, no. of comorbidities, insurance,
Wiley, 2021	United States; Cerner Real-World Data	Dec 2019 to Sep 2020	Anyone who tested for COVID-19 in the ED	Positive for COVID-19 in the ED	aOR: 0.94 (0.85, 1.08)	aOR: 0.99 (0.81, 1.21)	Insurance, age, sex, comorbidities, ICU admission, remdesivir, dexamethasone
Young, 2021	US Military; Armed Forces Health Surveillance Division	2020		Positive for COVID-19	aOR: 1.28 (1.08, 1.53)	aOR: 1.21 (1.01, 1.45)	Age, sex, rank, comorbidities, service branch, geographic region, occupation, marital status

Odds ratios shown with 95% CI. Abbreviations: aOR: adjusted odds ratio; aRR: adjusted risk ratio; BMI: body mass index; CCI: Charlson Comorbidity Index; COPD: chronic obstructive pulmonary disease; ED: emergency department; EHR: electronic health record; ICU: intensive care unit; SIP: stay in place.

Supplemental Table S3. Sample Characteristics: Hospital Patients Positive for COVID-19 from March 2020 through April 2021 (N = 19,241) Who Were Admitted vs. Not Admitted

Variable, n (%)	Not Admitted	Admitted[a]
	n = 15,926	n = 3,495
Age		
<35	6,205 (39.0)	314 (9.0)
35–50	4,857 (30.5)	658 (18.8)
50–65	3,480 (21.9)	1,083 (31.0)
65+	1,384 (8.7)	1,440 (41.2)
Male	6,600 (41.4)	1,764 (50.5)
Race and ethnicity		
Black	4,765 (29.9)	1,372 (39.3)
Hispanic	7,291 (45.8)	1,338 (38.3)
White	3,870 (24.3)	785 (22.5)
Insurance		
Commercial	8,540 (53.6)	1,041 (29.8)
Medicaid	2,821 (17.7)	663 (19.0)
Medicare	1,369 (8.6)	1,452 (41.6)
Uninsured	3,195 (20.1)	339 (9.7)
Has PCP	13,133 (83.6)	2,965 (85.3)
Had PCP visit in prior 24 months	5,285 (33.2)	1,303 (37.3)
Had any encounter in prior 12 months	6,871 (43.1)	3,235 (92.6)
ED encounter	4,524 (28.4)	
Test locations		
Drive-through	6,229 (39.1)	82 (2.4)
ED	4,488 (28.2)	3,318 (95.0)
Employee testing	237 (1.5)	0
PCP/Specialist clinic	235 (1.5)	10 (0.3)
Urgent/Convenient care	1,390 (8.7)	35 (1.0)
Other outpatient	3,347 (21.0)	50 (1.4)

[a] Chi-squared test of association used to compare being admitted vs. each variable. All variables were significant at p-value < 0.05.

Supplemental Figure S1 Flowchart of the Percentage of COVID-19 Positive Patients Admitted by Race and Ethnicity

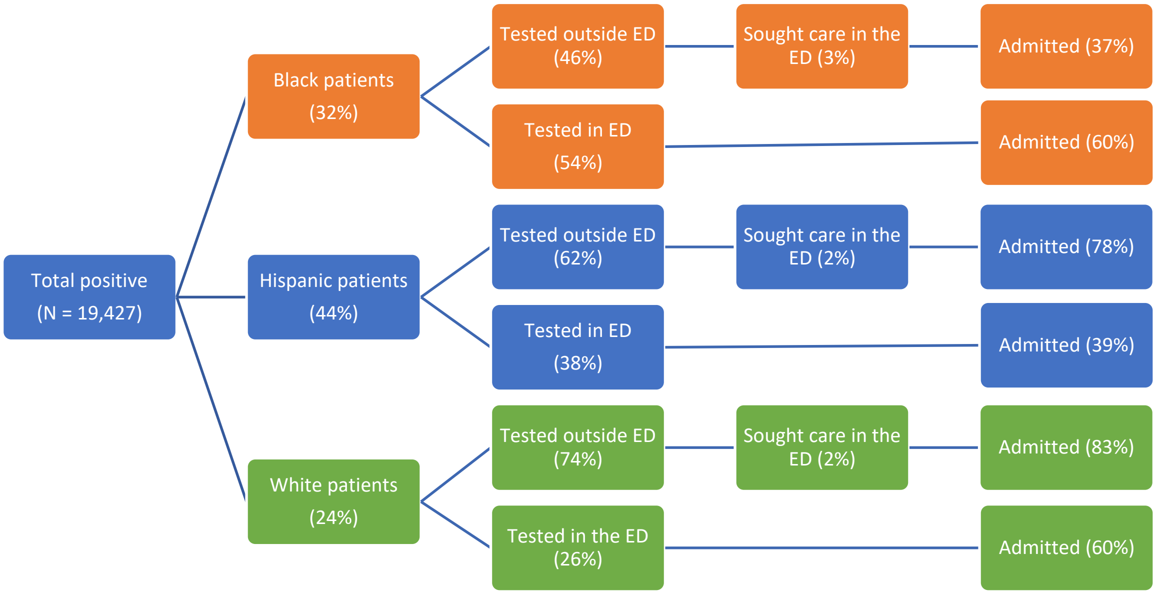

Percentage shown is the percentage out of the previous block. For example, 32% of all COVID-19 positive patients were Black, and 46% of all Black patients in the study were tested outside the ED.
